# Ogilvie's Syndrome after Cesarean Section: Case Report in Saudi Arabia and Management Approach

**DOI:** 10.1155/2017/5328160

**Published:** 2017-12-27

**Authors:** Lamiaa Elsebay, Mariam Ahmed Galal

**Affiliations:** ^1^Specialized Medical Center Hospital, Riyadh, Saudi Arabia; ^2^Alfaisal University, Riyadh, Saudi Arabia; ^3^SCFHS, Riyadh, Saudi Arabia

## Abstract

**Background:**

Acute colonic pseudoobstruction or Ogilvie's syndrome is a rare entity that is characterized by acute dilatation of the colon without any mechanical obstruction. It is usually associated with medical disease or surgery and rarely occurs spontaneously. If not diagnosed early, Ogilvie's syndrome may cause bowel ischemia and perforation.

**Case:**

A G7P4+2, 40-year-old woman, who is a known case of gestational diabetes mellitus during her current pregnancy, four previous cesarean sections, two early pregnancy losses at six-week gestation, and hypothyroidism, underwent uncomplicated elective cesarean section, after which she complained of abdominal distention.

**Conclusion:**

Ogilvie's syndrome is a rare condition yet of interest to obstetricians, midwifery staff, and general surgeons because its early diagnosis and prompt treatment are the keystones to avoid any subsequent fatal complications. This case report reviews the clinical characteristics, diagnostic methods, and management of Ogilvie's syndrome. Moreover, we suggest a management approach to help in early diagnosis and prompt management to improve the outcome of this potentially serious condition.

## 1. Introduction

The acute colonic pseudoobstruction (ACPO), nonobstructive colonic dilatation, or Ogilvie's syndrome is a rare entity that is characterized by acute dilatation of the colon, usually involving caecum and right hemicolon in the absence of any mechanical obstruction (80–90%), abdominal pain (80%), abdominal tenderness (62%), nausea and/or vomiting (60%), constipation (40%), and fever (37%). It is usually associated with an underlying illness, infection, or surgery and rarely occurs spontaneously. Identification of this condition is important due to the increased risk of subsequent bowel ischemia and perforation, particularly with caecal diameter >9 cm, with high mortality rate up to 50%. Here, we report a case of right colon necrosis and perforation after cesarean section that leads to urgent laparotomy and highlights early and appropriate diagnosis from an obstetric point of view.

## 2. Case Presentation

A 40-year-old female, G7P4+2, was admitted for elective cesarean section at 38 weeks. Her medical history included gestational diabetes mellitus (GDM) during her current pregnancy that was controlled on metformin (500 mg, three times daily), four previous cesarean sections, two early pregnancy losses at six-week gestation, hypothyroidism, and previous eye surgery at childhood for eye squint. Her family history was positive for diabetes and hypertension.

The patient had an elective cesarean section under spinal anesthesia and gave birth to a living female. It was noticed that she has been omental to the anterior abdominal wall adhesions and omental to the anterior uterine wall adhesions. There were no intraoperative complications and estimated blood loss was about 500 cc.

On the first postoperative day [POD1], the patient looked well with stable vital signs. System review was within normal, and physical examination showed soft and lax abdomen with audible bowel sounds. The patient was started on the liquid diet. The patient passed flatus and was started on the soft diet. The same day at night, she developed mild abdominal distension, bowel sounds still audible with stable vital signs, and the patient was advised to mobilize. The patient mentioned she used to have more abdominal distension after each caesarian delivery.

On POD2, the patient started to have more abdominal distension despite passing stool, and bowel sounds become sluggish then nonaudible. Patient was kept NPO; serum electrolytes were requested and showed mild hypokalemia 3.29 mmol/L. Patient was encouraged to mobilize and was started on potassium chloride infusion and NGT was inserted. She initially was diagnosed to have paralytic ileus, but her general condition eventually deteriorated dramatically, and she developed tachycardia and shortness of breath.

**Figure 1:**
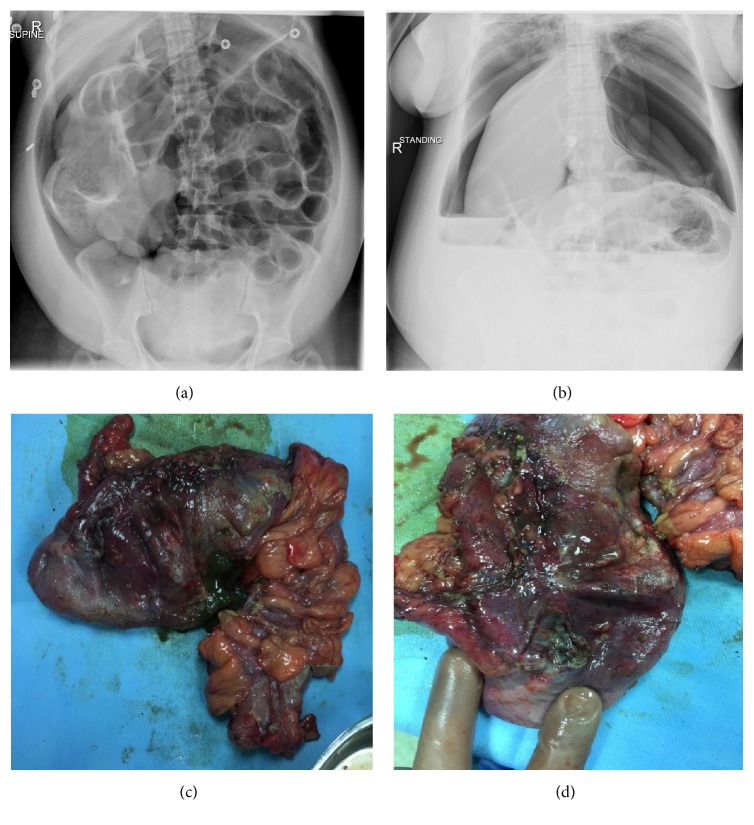


The patient was transferred to the Intensive Care Unit (ICU), reviewed by ICU and surgical team. Abdominal X-ray was performed and showed distended abdomen with pneumoperitoneum (see Figures 1(a) and 1(b)). CT scan was requested and showed small amount of free fluid collection in the subphrenic area with subhepatic longitudinal mass and large pneumoperitoneum suggesting possible bowel perforation and dilated proximal small bowel loops without obvious transitional zone. The patient was transferred to OR for exploration laparotomy.

Exploration laparotomy performed through longitudinal abdominal incision. There was gangrenous changes of the caecum and right colon with its anterior wall showing multiple ischemic areas and necrosis; some of them are perforated with gross picture of ischemic changes, others thinned out and were about to perforate in subhepatic area; right hemicolectomy and iliostomy were performed till the area of normal color of the colon was reached (see Figures 1(c) and 1(d)). Peritoneal lavage was performed afterwards, 2 abdominal drains were inserted, and the incision site was closed with staples. The patient was properly hydrated all through the surgery, fluid input/output were properly calculated, and urine output was adequate and clear. The uterus and both adnexa were normal.

The patient was transferred back to ICU. The patient received broad-spectrum antimicrobial agents; she was under close monitoring, multidisciplinary team management and discharged to regular room 6 days postoperatively. The postoperative course passed otherwise uneventful. The multidisciplinary team shared in plan of care were surgeons, pulmonologists, ICU intensivists, obstetricians, and cardiologist. Thrombophilia screening was suggested and hyperhomocysteinemia was found; homocysteine level was 14.26 Umol/L. She was discharged in a good general condition 12 days postoperatively.

## 3. Discussion

Ogilvie's syndrome or ACPO was first reported by Sir Ogilvie in 1948 [1]. It is described as acute dilatation of the colon usually involving caecum and right hemicolon without any existing mechanical obstruction [2, 3]. It is a rare condition yet it can result in dangerous complications with subsequent high mortality rate beyond 50% [4]. It can occur at any age with higher frequency in the sixth decade of life [5]. Its incidence in males is higher than that in females (1.5 : 1) [5].

It has been reported after pregnancy or cesarean section [6]. The condition has been also associated with trauma, severe burns, drugs (narcotic analgesics, antidepressants, corticosteroids, antipsychotic, calcium channel blockers, narcoleptics, and syntocinon), spinal anesthesia, opioid use, alcohol, cardiac failure, respiratory failure, neurological problems (Parkinson's, Multiple Sclerosis, and Alzheimer's), electrolyte imbalance, stress that causes central secretion of corticotrophin-releasing factor (which, in turn, inhibits gut motility), and hormones affecting the smooth muscles and, in rare occasions, may occur spontaneously [6–9]. The etiological factors in our case were as follows: (1) a multiparous patient; (2) previous repeated cesarean sections; (3) hormonal effect of pregnancy, GDM, and hypothyroidism; (4) receiving spinal anesthesia; and (5) age (40 years old).

The exact pathophysiology of the disease is still unclear but it was hypothesized that either the increase in the sympathetic tone or the decrease in the sacral parasympathetic innervations to the colon results in decreased colon motility with subsequent proximal colon dilation which will eventually increase the intraluminal pressure in the proximal colon and cecum, obstructing the caecal capillary circulation and causing subsequent ischemia, gangrene, and perforation [2, 8, 10]. This explanation is widely accepted due to the proximity between autonomic nerves and the structures at risk during cesarean section, including the cervix, the vagina, and the broad ligaments [6]. Regardless, the true pathogenesis of the syndrome is thought to be multifactorial.

As the ACPOs have serious complications, timely diagnosis and treatment are critical. Clinical and radiological findings are both needed to confirm the diagnosis of the syndrome [10]. Typically, it presents within 48h and up to 12days postoperatively and can be confused with mechanical obstruction of bowel-like paralytic ileus (see Table 1) [5, 11, 12]. Clinical features include abdominal distension with mild-to-moderate abdominal discomfort, constipation, nausea, and vomiting along with low grade fever [8, 13, 14]. Clinical examination may show mild-to-moderate tenderness with bowel sounds noted in 90% of patients [8, 10]. Abnormal bowel sounds reported were either hyperactive, high-pitched, or sometimes absent [12]. In our reported case, the clinical features included abdominal pain, distension, and vomiting.

Plain abdominal X-ray is the most useful diagnostic modality that reveals gaseous distention in colon, mostly involving the caecum and ascending colon, with or without fluid levels seen in small bowel [10, 11]. Caecal diameter of 9–12 cm warrants ischemia and subsequent perforation if not managed urgently [5]. Though X-ray is a fundamental diagnostic modality, other modalities like CT scans and water soluble contrast enema are used to confirm the diagnosis and to exclude mechanical obstruction [10]. When X-ray is indeterminate and the correct diagnosis cannot be made, gastrografin enema is desirable to detect bowel distention. However, an exploratory laparotomy in some patients will remain the final option to reach a conclusive diagnosis [13]. In our case, the initial diagnosis made was paralytic ileus and bowel perforation, yet the final diagnosis of Ogilvie's syndrome was reached only after laparotomy, when several areas of necrosis and perforation were seen.

In ACPO, laboratory findings are nondiagnostic. Some electrolyte imbalances like hyponatremia, hypomagnesemia, and hypokalemia can be seen in ACPO, but they represent a consequence of the pathological condition rather than its etiologic factor. Similarly, leukocytosis can be present, especially with perforation or bowel ischemia. Hypokalemia and leukocytosis were present in our case.

Management for uncomplicated patients is initially conservative with limiting oral intake, active mobilization, cessation of opioids, and correction of electrolytes, and underlying comorbidities should be treated [14]. Intravenous hydration, nasogastric decompression, rectal tube decompression, close clinical monitoring with serial physical examinations, laboratory studies, and abdominal radiological modalities should be done [5].

The most effective pharmacological agent is neostigmine, given intravenously at a dose of 2mg over 3–5min and repeated once if required in 2-3 hours [11]. Neostigmine is a reversible anticholinesterase inhibitor that potentiates the effects of the parasympathetic system and improves colonic motility, causing effective colonic decompression up to 88%. Ganglionic blockade with guanethidine followed by cholinergic stimulation with neostigmine can be effective. Neostigmine should not be used in overly distended caecum and, due to its bradycardiac hypotensive effects, it should be given to vitally stable patient with monitored setting [13]. An alternative to neostigmine is erythromycin, a motilin receptor agonist [13]. Other pharmacological agents are naloxone and cisapride [5].

If conservative and medical management, including the second dose of neostigmine, failed, colonoscopic decompression is recommended. It is successful in 68–95% of cases and prevents any ischemia and bowel perforation, yet recurrence is common. Colonoscopic decompression is contraindicated if perforation or peritonitis exists [6].

Surgery is recommended if colonoscopic decompression failed, or progressive clinical deterioration or signs of ischemia and perforation are present, or if caecal diameter is >12 cm. Surgical treatment can be either caecostomy or, in case of ischemic bowel, hemicolectomy with or without primary anastomosis or total abdominal colectomy. The surgical treatment has mortality rate ranging from 30% to 60%.

For pregnant woman with severe constipation and undergoing C-section, certain measures can be done preop and intra-op to prevent or reduce the occurrence of adynamic ileus (see Table 2) [7]. For postoperative pain control, nonsteroidal anti-inflammatory drugs may be considered in place of opioids for high-risk patients. Having similar pain relieving effect as systemic opiates, thoracolumbar epidural anesthesia can be used to reduce the duration of postoperative ileus.

**Figure 2:**
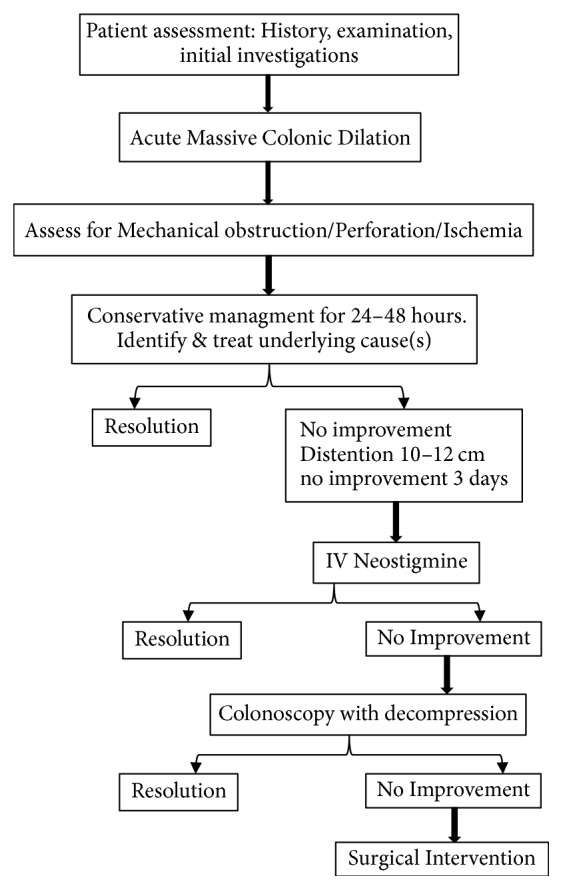
Stepwise approach to Ogilvie's syndrome.

After review of cases, we suggest the management algorithm (see Figure 2). Once the case is suspected (severe abdominal distention, abdominal pain, nausea, and constipations), it is required to obtain initial proper assessment including history (surgeries, caesarian section, infection, and spinal injuries, keeping in mind the previously discussed etiological factors), clinical examination, and laboratory investigations (absence of mechanical obstruction favor the diagnosis of Ogilvie's syndrome or ACPO). Conservative management should be established for 24–48 hours in the form of close observation, hydration and correction of electrolyte imbalance, insertion of the nasogastric tube, keeping the patient fasting without using rectal enema or laxatives, cessation of all narcotic medications, and treating underlying etiological factors if any. Assess to rule out the presence of mechanical obstruction and to evaluate for perforation as this will terminate conservative management. In the case of no improvement, when abdominal X-ray shows caecal dilation, colonic distention 10–12 cm, likely normal small bowel, and no mechanical obstruction or no improvement for 3 days, pharmacological decompression with intravenous neostigmine could be started with caution if cecum is significantly dilated and if there is no response, endoscopic colonic decompression can be considered. If pharmacological/endoscopic decompression failed or signs suggestive of perforation exist, then surgical intervention should be considered in the form of caecostomy, hemicolectomy, and resection of the ischemic or perforated segment of the bowel to be performed.

To conclude, Ogilvie's syndrome is rare yet very important to obstetricians, midwifery staff, and general surgeons to diagnose and manage it as early as possible in patients who underwent C-section to avoid any subsequent fatal complications. The authors recommend precise assessment and close monitoring with conservative management in any suspected case. Reassessment is important to assess whether the disease progresses or regresses. With progression, medical, interventional, and surgical management can be considered as described in the context.

## Figures and Tables

**Table 1:** Compares between Ogilvie's syndrome and paralytic ileus.

	Ogilvie's syndrome	Paralytic ileus
Impaired area	Limited to colon	Throughout the gut
Bowel sounds	Hyperactive/high-pitched/absent	Always absent
Nausea & vomiting	Mild and inconstantly present	More common
Passing flatus	Present	Always ceased
Passing stool	Present/diarrhea/obstipation	Always ceased

**Table 2:** Pre- and intraoperative measures taken in pregnant women to avoid adynamic ileus.

Preoperative measure	Intraoperative measure
Correct poor bowel habits during pregnancy Perform enema before CS	Reduce blood loss Early blood transfusion Maintain stable hemodynamic status Minimize operation time Avoid intestinal protrusion out of the abdominal cavity due to vomiting

## References

[1] Ogilvie H. (1948). Large-intestine colic due to sympathetic deprivation; A new clinical syndrome. *British Medical Journal*.

[2] Camilleri M. Acute Colonic Pseudo-Obstruction (Ogilvie's Syndrome). http://www.uptodate.com.

[3] Cebola M., Eddy E., Davis S., Chin-Lenn L. (2015). Acute colonic pseudo-obstruction (Ogilvie's syndrome) following total laparoscopic hysterectomy. *Journal of Minimally Invasive Gynecology*.

[4] Ponzano C., Nardi S., Carrieri P., Basili G. (1997). Diagnostic problems, pathogenetic hypothesis and therapeutic proposals in Ogilvie's syndrome. *Minerva Chirurgica*.

[5] Shakir A. J., Sajid M. S., Kianifard B., Baig M. K. (2011). Ogilvie's syndrome-related right colon perforation after cesarean section: A case series. *Kaohsiung Journal of Medical Sciences*.

[6] Saha A. K., Newman E., Giles M., Horgan K. (2009). Ogilvie's syndrome with caecal perforation after Caesarean section: A case report. *Journal of Medical Case Reports*.

[7] Cho F.-N., Liu C.-B., Li J.-Y., Chen S.-N., Yu K.-J. (2009). Adynamic Ileus and Acute Colonic Pseudo-obstruction Occurring After Cesarean Section in Patients With Massive Peripartum Hemorrhage. *Journal of the Chinese Medical Association*.

[8] Vanek V. W., Al-Salti M. (1986). Acute pseudo-obstruction of the colon (Ogilvie's syndrome). An analysis of 400 cases. *Diseases of the Colon & Rectum*.

[9] Dickson M. A. S., McClure J. H. (1994). Acute colonic pseudo-obstruction after caesarean section. *International Journal of Obstetric Anesthesia*.

[10] Alshareef R. Y. (2016). Pediatric acute colonic pseudo-obstruction post complicated appendicitis. *International Journal of Case Reports and Images (IJRCI)*.

[11] Latunde-Dada A. O., Alleemudder D. I., Webster D. P. (2013). Ogilvie's syndrome following caesarean section. *BMJ Case Reports*.

[12] Kalu E., Fakokunde A., Jesudason M., Whitlow B. (2005). Acute colonic pseudo-obstruction (Ogilvie's Syndrome) following caesarean section for triplets. *Journal of Obstetrics & Gynaecology*.

[13] Bhatti A. B., Khan F., Ahmed A. (2010). Acute colonic pseudo-obstruction (ACPO) after normal vaginal delivery. *J Pak Med Assoc*.

[14] Khajehnoori M., Nagra S. (2016). Acute colonic pseudo-obstruction (Ogilvie's syndrome) with caecal perforation after caesarean section. *Journal of Surgical Case Reports*.

